# Pentameric C-reactive protein is a better prognostic biomarker and remains elevated for longer than monomeric CRP in hospitalized patients with COVID-19

**DOI:** 10.3389/fimmu.2023.1259005

**Published:** 2023-09-01

**Authors:** Francis R. Hopkins, Johan Nordgren, Rafael Fernandez-Botran, Helena Enocsson, Melissa Govender, Cecilia Svanberg, Lennart Svensson, Marie Hagbom, Åsa Nilsdotter-Augustinsson, Sofia Nyström, Christopher Sjöwall, Johanna Sjöwall, Marie Larsson

**Affiliations:** ^1^ Division of Molecular Medicine and Virology, Department of Biomedical and Clinical Sciences, Linköping University, Linköping, Sweden; ^2^ Department of Pathology & Laboratory Medicine, University of Louisville, Louisville, KY, United States; ^3^ Division of Inflammation and Infection, Department of Biomedical and Clinical Sciences, Linköping University, Linköping, Sweden; ^4^ Division of Infectious Diseases, Department of Medicine, Karolinska Institute, Stockholm, Sweden; ^5^ Department of Infectious Diseases, Vrinnevi Hospital, Norrköping, Sweden; ^6^ Clinical Immunology and Transfusion Medicine, Department of Biomedical and Clinical Sciences, Linköping University, Linköping, Sweden

**Keywords:** CRP, COVID-19, SARS-CoV-2, isoforms, prognostic marker

## Abstract

The differing roles of the pentameric (p) and monomeric (m) C-reactive protein (CRP) isoforms in viral diseases are not fully understood, which was apparent during the COVID-19 pandemic regarding the clinical course of severe acute respiratory syndrome coronavirus 2 (SARS-CoV-2) infection. Herein, we investigated the predictive value of the pCRP and mCRP isoforms for COVID-19 severity in hospitalized patients and evaluated how the levels of the protein isoforms changed over time during and after acute illness. This study utilized samples from a well-characterized cohort of Swedish patients with SARS-CoV-2 infection, the majority of whom had known risk factors for severe COVID-19 and required hospitalization. The levels of pCRP were significantly raised in patients with severe COVID-19 and in contrast to mCRP the levels were significantly associated with disease severity. Additionally, the pCRP levels remained elevated for at least six weeks post inclusion, which was longer compared to the two weeks for mCRP. Our data indicates a low level of inflammation lasting for at least six weeks following COVID-19, which might indicate that the disease has an adverse effect on the immune system even after the viral infection is resolved. It is also clear that the current standard method of testing pCRP levels upon hospitalization is a useful marker for predicting disease severity and mCRP testing would not add any clinical relevance for patients with COVID-19.

## Introduction

Severe acute respiratory syndrome coronavirus 2 (SARS-CoV-2) is the agent of the COVID-19 pandemic and gives rise to mild or moderate symptoms in most infected individuals. However, 10-15% progress to severe disease with pneumonia, acute respiratory distress syndrome (ARDS), and multiple organ failure. SARS-CoV-2 infection activates innate and adaptive immune responses, which can lead to uncontrolled inflammation, a so called “cytokine storm”, which is advocated as a key pathogenetic factor in severe COVID-19 (1). Among the factors induced by the infection is the C-reactive protein (CRP), an acute-phase protein mainly produced in the liver by hepatocytes (2). While CRP levels are typically low in healthy individuals, the level of this protein can increase significantly in the presence of inflammation, making it a widely used and valuable diagnostic biomarker for e.g., inflammatory diseases such as rheumatoid arthritis (RA), and bacterial infections (3, 4).

CRP is initially produced and released into the bloodstream in its pentameric isoform (pCRP), which can irreversibly dissociate to a monomeric isoform (mCRP) at sites of inflammation. In an inflammatory setting the pCRP isoform binds phosphatidylcholine on the surface of microorganisms and damaged host cells and this leads to its degradation to mCRP (5). The two different isoforms of CRP exhibit distinct characteristics and can have both pro-inflammatory and anti-inflammatory functions depending on the specific disease context and cell types involved (6–9). The mCRP induces higher levels of inflammatory factors including nitric oxide, C-X-C motif chemokine ligand 8 (CXCL8), and monocyte chemoattractant protein 1 (MCP-1) in immune cells such as neutrophils compared to pCRP and support the recruitment of immune cells to areas of inflammation (10–12). The CRP isoforms can bind a variety of ligands, including complement component 1q (C1q), Fcγ-receptors (Fcγ-R), as well as nuclear antigens (13, 14). pCRP has been found to bind to both Fcγ-RI and Fcγ-RIIa, while mCRP has been suggested to bind with higher affinity to Fcγ-RIII (5). The different binding affinities of mCRP and pCRP to Fcγ-Rs may contribute to their differential functions and deposition on different immune cells. There are indications that pCRP is more potent at activating the complement system and promoting clearance of microorganisms by immune cells compared to mCRP (15). In addition, the ratio of pCRP to mCRP may be altered in certain disease states, such as sepsis and systemic lupus erythematosus, where an increase in mCRP levels has been observed (4, 7).

In patients with severe COVID-19 a significant increase in pCRP levels has been observed compared to patients with mild disease (16–19), and pCRP levels have been linked to increased mortality in COVID-19 (20). Indeed, elevated pCRP levels reflect the hyperinflammatory response, which is a major clinical manifestation of severe COVID-19 that might lead to severe lung damage and death (21). In addition, several of the elevated inflammatory markers in patients with COVID-19, e.g., interleukin-6 (IL-6), tumor necrosis factor (TNF), and ferritin have been found to correlate positively with pCRP levels (22). In a recent study mCRP levels were shown to independently associate with COVID-19 severity (23).

The aims of the current study were to determine if pCRP and mCRP levels are associated with COVID-19 disease severity to establish if they can be used as biomarkers, and to assess the levels of pCRP and mCRP over time in hospitalized patients with COVID-19. We found that while pCRP levels were higher in patients with severe/critical COVID-19, the mCRP levels were similar between patients with mild/moderate and severe/critical disease. Additionally, both pCRP and mCRP were increased at the two-week follow-up, with pCRP levels remaining elevated for at least six weeks, suggesting that the inflammation still is ongoing, likely due to the damage caused by the initial high level of inflammation triggered by the SARS-CoV-2 infection.

## Materials and methods

### Demographics and clinical characteristics of patients

Hospitalized COVID-19 patients (N=62) were included in the study from August 2020 to May 2021 as soon as possible following admission to the Department of Infectious Diseases at the Vrinnevi Hospital, Norrköping, Sweden. Healthy, SARS-CoV-2 RNA negative controls (N=31) were recruited among health care workers at the Vrinnevi Hospital, Norrköping, Sweden. The study protocol was approved by the Swedish Ethical Review Authority (Decision number 2020–02580). Oral and written informed consent was obtained from all participants.

At inclusion in the study, a panel of clinical markers, including CRP, lactate dehydrogenase (LDH), and numbers of neutrophils, monocytes, and lymphocytes, was assessed (**Table 1**). In addition, data concerning smoking habits, medication, body mass index (BMI), and co-morbidities such as diabetes, cardiovascular disease, renal failure, and chronic pulmonary disease was collected. The patients were divided into two groups based on disease severity and according to the NIH COVID-19 patient treatment criteria, including symptoms, oxygen saturation in room air, clinical findings, and chest imaging, and also taking into account the highest level of care (pandemic department, intermediate or intensive care unit). The first group included cases with mild/moderate disease (mild, without oxygen supplementation at pandemic department, and moderate with oxygen supplementation ≤5 L/min at pandemic department). The second group included cases with severe/critical disease (severe, with oxygen supplementation > 5 L/min supplemented by high-flow nasal oxygen (HFNO) or continuous positive airway pressure (CPAP) at pandemic department or intermediate care unit, and critical with treatment in intensive care unit with or without mechanical ventilator) (24).

**Table 1:** Clinical characteristics and analytical variables of hospitalized patients with COVID-19.

Variable	Total N=62	Mild/Moderate disease N=31	Severe/Critical disease N=31	P-value*
Age, years median (range)	57.5 (32-91)	59 (32-91)	57 (32-78)	0.526
Male sex, N (%)	41 (66)	20 (65)	21 (68)	0.788
Symptom duration, days median (range)	10 (2-30)	10 (2-24)	10 (5-30)	0.576
Length of hospital stay, days median (range)	7 (2-54)	6 (2-23)	10 (3-54)	<0.001*
Intensive care, N (%)	8 (13)	0	8 (26)	0.005*
Stay at intensive care unit, days median (range)	9 (1-24)	N/A	9 (1-24)	N/A
Remdesivir, N (%)	22 (35)	8 (26)	14 (45)	0.184
Corticosteroid therapy, N (%)	40 (65)	14 (45)	26 (84)	0.003*
Mechanical ventilation, N (%)	4 (6.5)	0	4 (13)	0.113
Deceased, N (%)	3 (4.8)	0	3 (9.7)	0.238
Pre-existing comorbidities				
Diabetes, N (%)	15 (24)	6 (19)	9 (29)	0.554
Cardiovascular disease, N (%)	35 (56)	14 (45)	21 (68)	0.124
Chronic pulmonary disease, N (%)	15 (24)	8 (26)	7 (23)	1
Current or ex-smoker, N (%)	34 (55)	17 (55)	17 (55)	1
Acute renal failure, N (%)	10 (16)	6 (19)	4 (13)	0.731
Chronic renal failure, N (%)	7 (11)	3 (9.7)	4 (13)	1
Renal replacement therapy, N (%)	3 (4.8)	0	3 (9.7)	0.238
Body mass index (kg/m^2^), median (range)	30 (22-45)	27.5 (22-43)	31 (24-45)	0.024*
Immunocompromised^#^ at inclusion, N (%)	8 (13)	5 (16)	3 (9.7)	0.449
Analytical variables				
Haemoglobin g/L	140 (90-171)	130 (90-149)	126 (97-171)	0.451
White blood cell count (x10^9^/L)	6.7 (0.4-49)	6.2 (0.4-17)	7.2 (1.4-49)	0.149
Platelet count (x10^9^/L)	239 (20-668)	234 (20-543)	239 (134-668)	0.475
Neutrophil count (x10^9^/L)	4.95 (0-17.5)	4.2 (0-15.6)	6.1 (0.9-17.5)	0.012*
Lymphocyte count (x10^9^/L)	1 (0.1-2.8)	1.2 (0.3-2.2)	0.9 (0.1-2.8)	0.089
Plasma creatinine (µmol/L)	69 (36-1224)	70 (40-390)	66 (36-1224)	0.46
eGFR MDRD (mL/min/1.73m²)	95 (4-95)	95 (10-95)	95 (4-95)	0.652
Lactate dehydrogenase (µkat/L)	6.45 (3.2-16)	5.6 (3.2-16)	7.2 (4-16)	0.002*
suPAR (ng/mL) median (range)	5.9 (2.9-29)	5.0 (2.9-11.1)	6.6 (4.2-29)^#^	0.002*
Neutrophil-lymphocyte ratio median (range)	4.9 (0.2-87.5)	3.7 (0.2-8.9)	6.1 (0.4-87.5)	0.002*
SARS-CoV-2 viral load (ct-value)^†^	35.9 (17.8-39.0)	39.0 (21.8-39.0)	32.2 (17.8-39.0)	0.015*
pCRP (µg/mL)	63 (6-477)	40 (6-376)	90 (10-477)	0.043*
mCRP (ng/mL)	33.02 (12.6-57.7)	32.72 (12.6-57.7)	33.3 (21.7-50.4)	0.736

N/A, Not applicable.

ct-value, cycle threshold-value.

*P ≤ 0.05 = significant. NR, not relevant, ^#^values missing for two patients, ^¤^values missing for two patients, ^&^values missing for one patient, ^†28^.

Additional clinically relevant data regarding the levels of soluble urokinase plasminogen activator receptor (suPAR) and viral load in nasopharynx samples were drawn from previously performed studies and used as clinical parameters (6, 25).

### pCRP and mCRP measurements

Serum samples from the hospitalized patients taken at inclusion 2-week, and 6-week visits, and from healthy controls, were assessed for pCRP and mCRP. Levels of pCRP were measured using a turbidimetry high sensitivity technique at the routine clinical chemistry department, at Linköping University Hospital.

Levels of mCRP were measured using an in-house sandwich enzyme-linked immunosorbent assay (ELISA) as previously described (7). Briefly, 96-well plates were coated overnight with a goat anti-human mCRP polyclonal antibody diluted in PBS and thereafter blocked overnight at 4°C with PBS containing 1% bovine serum albumin (PBS-BSA). Patient samples and standards of different concentrations of recombinant mCRP were added to the plate and incubated for 2h at room temperature. A mouse anti-human mCRP monoclonal antibody (8C10) diluted 1:200 in PBS-BSA was then added and incubated for 90min at room temperature, followed by incubation with a goat anti-mouse IgG antibody conjugated with horseradish peroxidase (Abcam (ab6789), Waltham, MA, USA). After an hour incubation at room temperature, substrate solution (3,3′,5,5′ tetramethylbenzidine) was added. The reaction was stopped using 1M H_2_SO_4_ and optical density measured at 450nm. Reagents for the mCRP ELISA (goat anti-human mCRP and the monoclonal 8C10 anti-mCRP antibody (26) were kindly provided by Drs. Lawrence Potempa and Ibrahim Rahab (Roosevelt University, Schaumburg, IL, USA).

### Statistical analyses

Statistical differences between pCRP and mCRP levels in the two severity groups were calculated in GraphPad Prism 9.4.0 using Mann-Whitney test. ANOVA with Dunn’s multiple comparisons test was performed using GraphPad Prism version 8.0 for Windows, GraphPad Software, San Diego, California USA, www.graphpad.com.

Univariate analysis was done either by Fisher’s exact test for binary variables or Mann-Whitney U test for continuous variables using SPSS version 27. Multivariate logistic regression was performed with the variables: BMI, suPAR, neutrophil to lymphocyte ratio, LDH, and viral load at inclusion, which were included in the model after being associated with disease severity at p < 0.1 level after univariate analysis (6).

## Results

### Clinical characteristics of COVID-19 patients

A total of 62 hospitalized patients with COVID-19 were included in this study. They comprised 41 males and 21 females, with a median age of 57.5 years. The two severity groups (mild/moderate and severe/critical) were of equal size (N=31). Length of hospital stay, corticosteroid therapy, body mass index, neutrophil count, NLR, suPAR, viral load, and LDH levels differed significantly between the groups (**Table 1**). The median age in the control group was 45 years old. The cohort is well characterized (6, 25, 27, 28) and reflects characteristics and co-morbidities commonly seen in hospitalized patients with COVID-19.

### Hospitalized patients with severe/critical COVID-19 had significantly higher levels of pCRP at inclusion compared to mild/moderate disease

**Figure 1:**
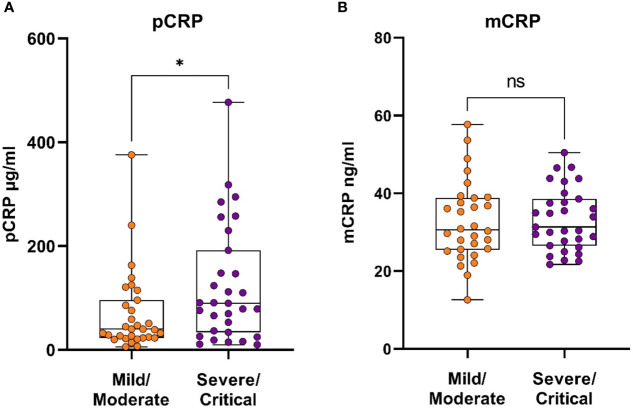
CRP isoforms in patients with COVID-19, stratified based on disease severity. Serum samples were taken from COVID-19 patients (N=62) upon hospitalization and assayed to determine the levels of circulating pCRP **(A)** and mCRP **(B)**. Mann-Whitney U test was used for statistical comparison. *P ≤ 0.05, ns, non-significant.

The levels of pCRP and mCRP were measured in blood samples taken from hospitalized patients with COVID-19 at inclusion (**Figure 1**). pCRP exhibited a significant difference between mild/moderate and severe/critical disease (**Figure 1A**), whereas there was no difference in mCRP levels between the severity groups (**Figure 1B**). Next, we performed a multivariate analysis using variables that associated in a univariate analysis; BMI (aOR 1.23, p=0.011), suPAR (aOR 1.47, p=0.046), neutrophil to lymphocyte ratio (aOR 1.074, p=0.29), LDH (aOR 1.2, p=0.23), and viral load; (aOR 7.04, p=0.054 for Ct value <30). pCRP did not associate with disease severity (adjusted odds ratio (aOR) 1.003, p=0.53) in this multivariate model. Nonetheless, comparing pCRP levels against only COVID-19 severity showed pCRP levels in COVID-19 patients to be a predictor for disease severity, supporting the use of pCRP as a biomarker.

### Persistence of elevated pCRP levels for at least 6 weeks in hospitalized patients with COVID-19

**Figure 2:**
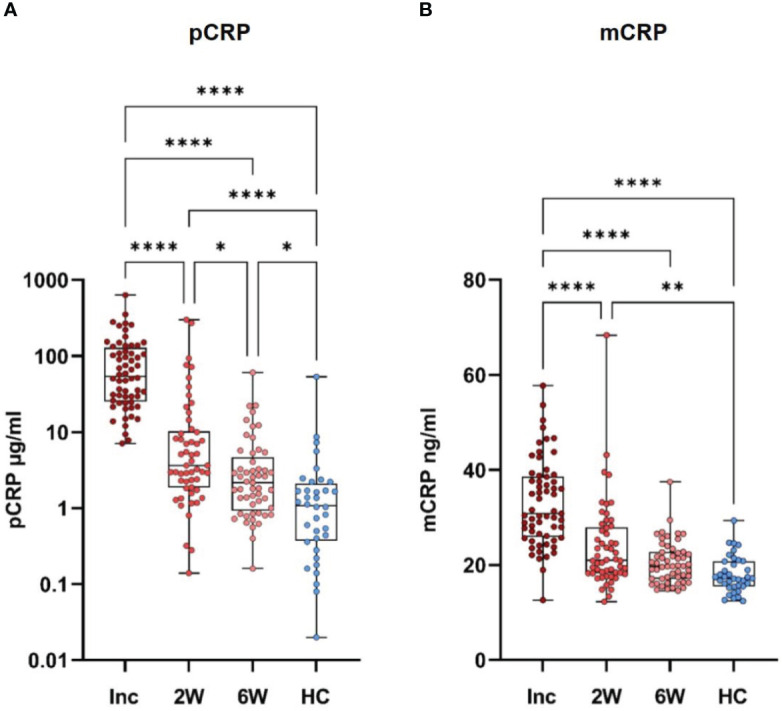
CRP isoforms are sustained for different length of time following hospitalization for COVID-19. Serum samples were taken from healthy controls (N=31) and COVID-19 patients at hospitalization (N=62). Follow up samples were taken at 2 and 6 weeks. All samples were assayed for pCRP **(A)** and mCRP **(B)**. Statistical testing was done by one way ANOVA with Dunn’s multiple comparisons test. *P ≤ 0.05, **P ≤ 0.01, ****P ≤ 0.0001. Inc, inclusion; 2W, 2 weeks; 6W, 6 weeks; HC, healthy controls.

Levels of pCRP and mCRP in serum samples collected from COVID-19 patients at inclusion, and at the 2-week, and 6-week follow-up were measured by turbidimetry high sensitivity assay and ELISA, respectively. pCRP levels in patients with COVID-19 were significantly increased at inclusion compared to healthy controls (**Figure 2A**). The pCRP levels decreased significantly between the inclusion and 2-week timepoint, and also between the 2-week and 6-week timepoints. Of note, pCRP levels were still significantly higher at the 6-week timepoint compared to the controls (**Figure 2A**). There were also significantly higher mCRP levels at inclusion and the 2-week time points compared to the healthy controls despite a significant drop between these two timepoints (**Figure 2B**). The mCRP levels had reached the level of healthy controls at the 6-week timepoint (**Figure 2B**). Although mCRP levels were significantly elevated as seen for pCRP, they returned more quickly to normal levels in circulation.

## Discussion

During the COVID-19 pandemic numerous biomarkers were evaluated for their ability to predict the severity of the SARS-CoV-2 infection. Here we investigated the pCRP and mCRP levels within a cohort of hospitalized COVID-19 patients throughout 2020 and 2021 in Sweden, to determine if both isoforms of CRP could be useful prognostic indicators for serious disease. We found pCRP but not mCRP levels were associated with COVID-19 disease severity, and the pCRP data is in line with multiple previous studies (16–19). Our mCRP data is not in line with a recently published study (23), in which mCRP was shown to have a better prognostic value for COVID-19 severity than pCRP.

Although pCRP is mostly considered to be a marker of inflammation, bacterial infection or sepsis, it was established early on that pCRP levels increase during SARS-CoV-2 infection (17, 18, 20, 23). Persistent elevated plasma levels of pCRP are found in chronic inflammatory diseases such as rheumatoid arthritis (3), type 2 diabetes mellitus, and Parkinson’s disease (29), and in chronic obstructive pulmonary disease (30). Whether mCRP levels are elevated in these diseases is not known, but mCRP has been implicated to play a pathogenic role in cardiovascular disease where it is detected in atherosclerotic plaques, and to cause neuroinflammation where it is found in the affected neuronal tissue (31–33). Consequently, mCRP is likely deposited in the inflamed lung tissue in patients with severe COVID-19. Therefore, in circulation, pCRP with its dynamic range is a better biomarker for COVID-19, whereas mCRP could be of more relevance to measure in the inflamed airway.

Of note, the elevation of pCRP following SARS-CoV-2 infection was sustained for a longer time compared to mCRP. Elevated pCRP levels are observed after acute COVID-19 infection, in people suffering from so-called ‘Long COVID’ (34), and our data here suggests that they are elevated even in convalescent people who do not have that specific syndrome. We also found a long-term elevation of circulating pCRP following the resolution of the infection and healing of the airways. We have previously shown that immune cells such as T cells, dendritic cells and monocyte subsets are affected up to 6 months after COVID-19, with an ongoing low level of inflammation, probably due to tissue damage and repair of the airways (27, 28). The elevated levels of pCRP may be a part in this ongoing inflammation, and further research should elucidate more of the mechanisms behind this. Work is also needed to clarify if mCRP plays any role in the ongoing inflammation, as one would expect from a modulator of inflammatory responses. It is of interest that the elevation of pCRP is more prolonged than that of mCRP, suggesting that the post-COVID immune environment is supportive for the production of pCRP but there is less dissociation into its monomeric form.

We used the National Institute of Health (NIH), USA COVID-19 patient treatment criteria (24) to stratify our patients according to disease severity, which could be one reason for the lack of association between mCRP levels and severity of COVID-19 in our study compared to the findings by Molins et al, which classified severe disease as intensive care admission and/or in-hospital mortality (23). Seeing that there were few mild and critical ill cases among our hospitalized COVID-19 patients, including patients might have improved the predictive power of mCRP, as similar studies used a cohort with a greater proportion of patients with mild and fatal disease (23). Additionally, it would have been of interest to investigate a wider range of inflammatory markers including cytokines. The cytokine storm is a well-described aspect of a severe SARS-CoV-2 infection and cytokines such as IL-6 and TNF have been shown to correlate to COVID-19 severity (22, 35). To date, there are no commercially available mCRP tests that can be employed efficiently in the clinical routine blood testing. Considering this, and the lack of correlation between mCRP and disease severity in our study, it appears unlikely that mCRP will make a better diagnostic test or be more useful as biomarker than pCRP in the clinical setting. In conclusion, in a cohort of hospitalized patients with COVID-19 we found that the inflammation, as shown by elevated pCRP levels, lasted for more than 6 weeks after SARS-CoV-2 infection. This indicates that COVID-19 gives rise to adverse effects on the immune system that last even after the viral infection has resolved. It is also clear that the clinical pCRP testing of COVID-19 patients upon hospitalization is a useful biomarker for predicting COVID-19 severity, as has been demonstrated here and by several studies (16–19) and would not be improved by additional analysis of mCRP levels.

## Data availability statement

The raw data supporting the conclusions of this article will be made available by the authors, without undue reservation.

## Ethics statement

The studies involving humans were approved by Swedish Ethical Review Authority Decision number 2020–0258. The studies were conducted in accordance with the local legislation and institutional requirements. The participants provided their written informed consent to participate in this study.

## Author contributions

FH: Data curation, Formal Analysis, Investigation, Visualization, Writing – original draft. JN: Data curation, Formal Analysis, Visualization, Writing – review & editing. RF-B: Investigation, Resources, Writing – review & editing. HE: Methodology, Writing – review & editing. MG: Writing – review & editing. CeS: Writing – review & editing. LS: Funding acquisition,Writing – review & editing. MH: Writing – review & editing. ÅN-A: Writing – review & editing. SN: Writing – review & editing. ChS: Conceptualization, Data curation, Funding acquisition, Methodology, Resources, Writing – review & editing. JS: Conceptualization, Data curation, Funding acquisition, Methodology, Resources, Writing – review & editing. ML: Conceptualization, Data curation, Funding acquisition, Methodology, Project administration, Supervision, Writing – original draft, Writing – review & editing.
